# Proliferative Vitreoretinopathy after Eye Injuries: An Overexpression of Growth Factors and Cytokines Leading to a Retinal Keloid

**DOI:** 10.1155/2013/269787

**Published:** 2013-09-30

**Authors:** Francesco Morescalchi, Sarah Duse, Elena Gambicorti, Mario R. Romano, Ciro Costagliola, Francesco Semeraro

**Affiliations:** ^1^Ophthalmology Clinic, Spedali Civili di Brescia, Department of Medical and Surgical Specialties, Radiological Specialties and Public Health, University of Brescia, 1 Piazzale Spedali Civili, Brescia 25123, Italy; ^2^Ophthalmology Clinic, Department of Health Science, University of Molise, Campobasso 86100, Italy; ^3^Ophthalmology Clinic, Istituto Clinico e di Ricerca Humanitas, Rozzano 86100, Milan, Italy

## Abstract

Eye injury is a significant disabling worldwide health problem. Proliferative Vitreoretinopathy (PVR) is a common complication that develops in up to 40–60% of patients with an open-globe injury. Our knowledge about the pathogenesis of PVR has improved in the last decades. It seems that the introduction of immune cells into the vitreous, like in penetrating ocular trauma, triggers the production of growth factors and cytokines that come in contact with intra-retinal cells, like Müller cells and RPE cells. Growth factors and cytokines drive the cellular responses leading to PVR's development. Knowledge of the pathobiological and pathophysiological mechanisms involved in posttraumatic PVR is increasing the possibilities of management, and it is hoped that in the future our treatment strategies will evolve, in particular adopting a multidrug approach, and become even more effective in vision recovery. This paper reviews the current literature and clinical trial data on the pathogenesis of PVR and its correlation with ocular trauma and describes the biochemical/molecular events that will be fundamental for the development of novel treatment strategies. This literature review included PubMed articles published from 1979 through 2013. Only studies written in English were included.

## 1. Introduction

Eye injury is a significant health problem worldwide that often results in disability; the National Research Council reported eye injury as the most underrecognized major health problem affecting those living in industrialized countries. There are approximately 203,000 cases of open-globe injury each year [1]. Such ocular trauma is the major cause of vision loss in young adults and children [2].

Up to 14% of ocular traumatic injuries result in severe vision loss or permanent blindness. It has been estimated that up to 19 million people are unilaterally blind as a result of ocular trauma. The high incidence of ocular trauma has extensive socioeconomic costs [2, 3]. Trauma can involve open- or closed-globe injuries, due to damage from sharp or blunt objects. Open injuries are classified in 4 subgroups on the basis of the type of trauma: rupture, penetration, perforation, and intraocular foreign body (IOFB). Closed-globe injuries are divided into 2 subgroups: contusion and laceration [4, 5].

Penetrating trauma is the most common cause of ocular morbidity; it is estimated that as many as 40% of globe penetration injuries are associated with retained IOFB [6–9]. The risk of visual loss is increased if the force that caused a closed-globe injury was sufficient to rupture the globe. Retinal detachment (RD) is a frequent sequel of severe ocular trauma, and RD often leads to proliferative vitreoretinopathy (PVR) [10, 11]. PVR is a complex cellular process characterized by the proliferation of membranes on or beneath the retina, intraretinal degeneration, gliosis, and contraction [12, 13]. By a mechanism not yet fully understood, excessive inflammation interferes with physiologic wound healing. This gives rise to an abnormal, protracted course of wound healing. Contraction of these proliferative membranes over the ultraspecialized tissue of the retina has disastrous consequences for vision.

PVR develops as a relatively rare complication in about 8–10% of patients with primary retinal detachment. The condition is much more frequent after trauma, occurring in 40–60% of patients with open-globe injury [14]. The frequency of PVR following perforation, rupture, penetration, persistence of an intraocular foreign body, and contusion is estimated to be 43%, 21%, 15%, 11%, and 1%, respectively [15]. 

The high incidence of PVR after ocular trauma is thought to be due to the inflammatory reaction that follows injury, which may have involved the direct introduction of cells from outside the eye. Those eyes that develop PVR after a trauma have worse visual outcomes, with PVR considered as the primary reason for the loss of vision [16].

In this review, we have summarized current knowledge on the pathogenesis of PVR and its correlation with ocular trauma and discussed how a fundamental understanding of the biochemical/molecular events involved is instrumental in developing novel treatment strategies.

## 2. Etiopathogenesis of PVR

Trauma to the retina gives rise to inflammation, which involves breakdown of the blood-retinal barrier (BRB). This process allows the body to heal and repair any tissue damage. Physiologic ocular wound healing involves inflammation, scar proliferation and modulation, tissue remodeling, and restoration of retinal integrity. This healing process includes the chemotaxis of inflammatory cells such as macrophages, lymphocytes, and polymorphonuclear cells and rarely evolves to PVR. However, when certain pathological events occur simultaneously, the stimulus to protracted wound healing triggers PVR. The most important of such events are retinal break, RD, and intravitreal hemorrhage. 

A retinal break is likely necessary for PVR; protracted exudative RD and hemivitreal detachments without holes are insufficient to trigger PVR [17]. The formation of a retinal break exposes the RPE to the vitreous cavity and its components, which leads to RD. The dimensions of the retinal break are directly and strongly correlated to the probability of PVR; giant retinal tears (width > 1 quadrant) are almost invariably followed by PVR. Rhegmatogenous RD occurs when the tractional forces of the vitreous on the retinal tear permit the fluid from the vitreous humor to enter the subretinal space (SRS). Vitreous fluid contains a large amount of cytokines and growth factors that stimulate the activation and the proliferation RPE and retinal glial cells [12, 18].

Once the retina has separated from the RPE, the increased distance to the choroidal blood supply and the reduced oxygen flux from the choriocapillaris to the photoreceptors lead to hypoxia. The resulting ischemia further compromises the BRB. Photoreceptors consume almost 100% of the oxygen provided to the retina by the choroid. An RD of only 1 mm creates sufficient hypoxia [19] to recruit proinflammatory cytokines to the RPE monolayer. Separation of the sensory retina from the underlying RPE violates the integrity of the tight junctions that form the BRB, which results in a loss of contact inhibition between RPE cells. These cells then grow in an uncontrolled manner into the vitreous.

The formation of a retinal tear or ocular injury can also trigger an intraocular hemorrhage. The direct influx of blood, serum proteins, and vitreal cells through the retinal break further stimulates PVR development. Research in animal models has shown that a single injection of fibroblasts was sufficient to induce PVR. Notably, the introduction of a sufficient amount of any cell type (whether macrophages, dermal cells, fibroblasts, or RPE cells) to the vitreous cavity results in pathology that mimics PVR. After a penetrating trauma, cells introduced from outside the eye (e.g., Tenon's layer or dermal tissue) may directly initiate PVR formation [20]. 

Inflammation, ischemia, and blood activate inflammatory cells (mainly macrophages, lymphocytes, and polymorphonuclear cells), which trigger the development of PVR through the formation of cytokines and growth factors [13]. Growth factors, cytokines, and proteins entering the SRS from the circulation come in direct contact with the RPE and glial or Müller cells, stimulating their proliferation.

### 2.1. Predisposing Factors

Several risk factors for developing PVR have been identified: size of the retinal hole or tear (cumulative break area > 3 optic discs), detachment involving > 2 quadrants, intraocular inflammation, vitreal hemorrhage, and preoperative choroidal detachment. 

Other predisposing factors are grade A or B preoperative PVR, the duration of RD before corrective surgery, high levels of vitreal proteins, repeated intraocular surgeries, aphakia, previous cryotherapy and photocoagulation, and the use of intraocular gas and silicone [21–23].

Kuhn and colleagues identified and stratified rupture, endophthalmitis, perforating injury, retinal detachment, and afferent pupillary defects as key risk factors predictive of a worse visual prognosis [24]. Additional risk factors for worse final best-corrected visual acuity (<20/40) are age (young patients, especially <5 years old), injuries with retrolimbal involvement, wound length ≥ 6 mm, and blunt injuries [25]. More posterior or longer wounds are also more likely to result in PVR. Vitreous hemorrhage is also strongly linked to less favorable outcomes [25–31].

The time from injury to the onset of PVR ranges from 1 to 6 months. A shorter interval between injury and PVR onset is observed for perforated globes (median, 1.3 months) followed by rupture (2.1 months), IOFB (3.1 months), penetration (3.2 months), and contusion (5.7 months) [15, 17].

### 2.2. Histopathology of RD and the Implications for PVR and Visual Outcomes

Adhesion of the neurosensory retina to the RPE is weak owing to the existence of a specialized extracellular SRS, in which the apical processes of the RPE interdigitate with the rod outer segments and specialized projections from the RPE ensheath the cone outer segments. This physiology stems from events during ontogenesis, when invagination of the optic vesicle into 2 layers forms the optic cup. The inner layer of the optic cup will eventually form the neuroretina, and the outer layer of the optic cup will form the RPE. Only pressure keeps the 2 layers apposed; the virtual space between them may be readily widened under the influence of weak tractional vitreous forces. Each RPE cell makes contact with 30–40 photoreceptors, forming a functional unit; survival of the photoreceptors is dependent on the RPE and vice versa [31, 32].

The RPE also contributes to the formation of the BRB, which in addition to maintaining ionic homeostasis of the SRS prevents proteins and blood components from penetrating neurosensory retina.

Anatomically, the neuroretina is usually considered to consist of 2 parts: the outer retina (which is avascular) and the inner retina (which is supplied with blood). The outer part is mainly nourished by diffusion from the choroid, while the inner half is supplied by the retinal circulation. Separation of the sensory retina from the underlying RPE deprives the outer retina of nutrients, with disruptive metabolic and neurochemical consequences for the entire retina. Most of detachment-induced retinal damage appears to be directly related to the reduced supply of oxygen and, to some extent, also to low levels of other substances, such as glucose [33–35]. The photoreceptor layer is by far the most vulnerable area, probably because the inner segments of the photoreceptors account for almost all oxygen consumption by the outer retina and because the outer retina is mainly supplied with oxygen and nutrients via diffusion from the choroid [36].

An RD alters the RPE-photoreceptor relationship [37, 38]. The outer retina becomes hypoxic [34]; the photoreceptors are stressed, and some die by apoptosis [39]. This is followed by programmed deconstruction of the surviving photoreceptor cells. A few hours or days after the RD, important cellular remodeling may be observed [40]. In the detached retina, the light-sensitive outer segments of rod photoreceptors degenerate and the synaptic terminals retract from the outer plexiform layer (OPL), so that rod synapses now occur deep in the outer nuclear layer (ONL). After a few days, up to 20% of photoreceptors (mainly rods) are apoptotic, while the other photoreceptors may have survived through changes in shape and/or metabolism but risk engulfment by the hypertrophic lateral branches of Müller cells. Müller glial cells, with their main stalk of cytoplasm extending across the width of the entire retina, undergo several changes in morphology during their lifespan [40]. Their nucleus often migrates into the ONL, at which point their main process and fine lateral branches increase in size and fill with glial fibrillary acidic proteins (GFAP) (intermediate filaments that play a role in mitosis).

Müller cells proliferate as part of an inflammatory response designed to heal the retina to protect neurosensory retina from mechanical stimuli (i.e., passive movement of the detached retina) and to protect photoreceptors from apoptosis. Müller cell proliferation is evident even in portions of the retina that are not yet detached, which suggests that RD involves a general reaction of the entire retina. Recent research seems to suggest that the release of diffusible growth factors such as PDGF from the site of retinal detachment induces the activation of Müller and glial cells, even in parts of the retina that remain attached [41].

However, hypertrophic Müller cells tend to fill all the empty spaces previously occupied by neurons that have degenerated, thus irreversibly altering retinal structure and function. In detached retina, the main stalk of the Müller cell often grows onto the surface of the ONL, along the outer limiting membrane and into the SRS where it can form a “glial scar.” Microglial cell proliferation and immune cell invasion may be detected in both detached and attached retinal areas. This proliferation contributes to retinal gliotic remodeling and to neuronal retinal degeneration, which could explain the impaired recovery of vision after reattachment surgery, particularly in patients with PVR [42].

Reattachment allows for the regrowth of outer segments and rod axons, although some of these now grow past the OPL, their normal target layer, and penetrate the inner retina. Reattachment inhibits the hypertrophy of Müller cells within the retina and in the SRS but appears to allow the growth of these cells onto the vitreal surface of the ganglion cell layer (GCL), where they form epiretinal membranes. Neuritic sprouts from the GCL often intermingle with the Müller cell processes that form epiretinal membranes. These protracted remodeling events, associated with photoreceptor cell death, often prevent complete functional recovery after surgical retinal reattachment.

Early reattachment probably halts and partially reverses the remodeling process and may stimulate withdrawal of many of the neurites that grow from these cells during detachment. However, prolonged detachment may stimulate further growth of Müller cells [41–43]. 

Restoration of the blood supply to the outer retina via reconnection with RPE microvilli stimulates the regrowth of outer segments and thus restores the retina's structural integrity [44]. It is reasonable to think that retinal reattachment represents a return of the retina to its “normal” state, but data from animal models suggests otherwise.

Reattachment has the ability to stop the growth of Müller cell processes into the SRS [42] but cannot stop growth in the opposite direction, which stimulates the formation of epiretinal membranes [41–43]. Müller cell changes allow for the formation of a scaffold that permits the adhesion and subsequent proliferation of other glial cells, leading to subretinal fibrosis and PVR.

Research performed in animal models suggests that one of the mechanisms by which Müller cells play a role in PVR is by upregulating the expression of PDGFR-*α* and GFAP, thus starting a process of dedifferentiation in cells whose behavior resembles that of fibroblasts [45]. Moreover, Müller cells in peripheral retina, where PVR most often occurs, have been shown to express stem cell markers indicative of active proliferation and dedifferentiation [46]. In addition, as yet unidentified cytokines and cofactors produced by migrated RPE cells may stimulate Müller cells to transform into cells with fibroblastic behavior, which then contribute to membrane formation and contraction. A thorough understanding of the molecular mechanisms underlying RD will be critical to controlling conditions such as PVR and may also elucidate associated rod axon outgrowth.

## 3. Pathobiology and Pathophysiology of PVR

Five distinct stages appear to be important in PVR development. These include breakdown of the BRB, chemotaxis and cellular migration, cellular proliferation, membrane formation with remodeling of the extracellular matrix, and contraction [47].

Soon after an RD, macrophages enter the vitreous cavity through the retinal injury [48, 49] and release inflammatory cytokines that stimulate cell migration and proliferation. However, immunohistochemical studies of PVR membranes show the presence of various subtypes of immune cells: macrophages, monocytes, T lymphocytes, B lymphocytes, glial cells, and cells expressing HLA-DR and DQ [50]. Macrophages and other inflammatory cells likely initiate the central event in the pathogenesis of PVR: the vigorous proliferation of RPE. Notably, the RPE is a monolayer of differentiated cells located between the neural retina and the choroidal vasculature essential for the survival of retinal neurons and visual function. The RPE contributes to the BRB, which, in addition to maintaining the ionic homeostasis of the SRS, prevents proteins and blood components from penetrating neural retina. The RPE is necessary for the preservation of normal photoreceptors and choriocapillaris and also plays an important role in the intraocular wound-healing response [51]. 

RPE cells are mitotically inactive under physiological conditions. Contact between the RPE and vitreous cytokines triggers dedifferentiation and epithelial-to-mesenchymal transformations.

Various signals have been found to trigger the migration and proliferation of RPE cells: the loss of contact, factors present in the vitreous, and signals from photoreceptors and inflammatory cells. Although RPE cells express receptors for hepatocyte growth factor (HGF), platelet-derived growth factor (PDGF), tumor necrosis factor (TNF), and other growth factors [52, 53], the interactions between RPE and Müller cells are likely the primary force regulating membrane formation and contraction [45].

Müller and RPE cell interaction can lead to the upregulation of PDGF-receptor *α* and increase Müller cell pathogenicity. Müller cells may also play a more active role than previously thought in the development of PVR membranes, especially when stimulated by an environment rich in RPE cells [46]. Depending on the size and age of the detachment as well as the size and location of the retinal tear, RPE cells are more or less likely to abandon their natural monolayer and migrate into the subretinal and preretinal space. These cells often attach to the vitreous, which acts as a scaffold, then migrate and secrete cytokines and cofactors that can alter Müller cell phenotype in ways that increase fibroblastic behavior and pathogenicity. 

BRB breakdown and blood coagulation over a wound site expose the RPE to various serum components, including thrombin, fibrin, and plasmin. Thrombin and fibrin have been shown to promote growth factor secretion, neural cell survival and apoptosis, cytoskeletal rearrangement, and cell proliferation [54]. Plasmin is a serine protease that dissolves fibrin blood clots. Plasmin has also been identified as the major PDGF-C processing protease in the vitreous of animal models of PVR as well as patients undergoing retinal surgery. Blocking plasmin may prevent the generation of active PDGF-C, the PDGF isoform most relevant to PVR. For this reason, plasmin was identified as a novel therapeutic target for patients with PVR [55].

RPE cells undergo an epithelial-mesenchymal transition [55–57] and develop the ability to migrate out into the vitreous, producing a provisional extracellular matrix containing collagen, fibronectin, thrombospondin, and other matrix proteins [58]. During this process, subretinal RPE cells may lose their connection to the RPE extracellular matrix [59–62] and migrate through the retinal break to enter the vitreous cavity. 

Kiilgaard et al. [63] used 5-bromo-2-deoxyuridine (BrdU) to detect proliferating RPE cells and found that posterior pole injury in the porcine eye results in RPE proliferation in the anterior part of the RPE but not in the vicinity of the lesion. This suggests that a population of RPE progenitor cells exists in the vicinity of the ora serrata [64]. These cells as well as the neural progenitors of Müller cells could supply the cells necessary for proliferation in PVR [46]. Notably, most PVR membranes are formed by fibroblasts. Animal models of PVR are typically created by injecting fibroblasts directly into the vitreous. The intravitreal fibroblasts observed in PVR derive ontologically from trans-differentiated RPE or Müller cells in the case of a primary rhegmatogenous RD and from fibroblasts that originated extraocularly in the case of ocular injury. 

The mechanisms of induction of posttraumatic PVR are probably the same implied in experimental PVR, obtained by injection of extraocular cells into the vitreous of animal models.

When a wound is created, membranes are often seen to extend intraocularly from the wound edge; the fibroblasts that constitute these membranes may be derived from Tenon's layer [10]. Fibroblasts and transdifferentiated cells give rise to myofibroblasts that bestow PVR membrane contractility. The contraction of these cells is responsible for the most deleterious effects of PVR, including retinal wrinkling and distortion, formation of new retinal breaks, and reopening of previously sealed breaks [65]. 

Two mechanisms have been proposed to explain the membrane contraction that can lead to a secondary RD. One is the active contraction of myofibroblastic cells; the second is the motile activity of myofibroblasts, which remodel the surrounding extracellular matrix [66]. The second mechanism is supported more strongly by scientific evidence. According to this theory, TGF-**β** secreted by macrophages induces the transformation of fibroblasts into smooth muscle- (SM-) actin-positive myofibroblasts [67]. 

### 3.1. Cytokines Involved in PVR

The emerging hypotheses regarding the pathogenesis of PVR have focused on abnormal local concentrations of growth factors and cytokines in the vitreous. This environment is conducive to transdifferentiation, migration, proliferation, survival, and extracellular matrix formation [68]. The growth factors likely to be involved are PDGF, TNF-**α** and TNF-**β**, HGF, transforming growth factor beta 2 (TGF*β*_2_), epidermal growth factor (EGF), and fibroblast growth factor (FGF). Cytokines such as interleukin- (IL-) 1, IL-6, IL-8, IL-10, and interferon gamma (INF-**γ**) are also thought to play a role. Recent experiments have focused attention on the activation of a receptor for PDGF (PDGFR-**α**), which seems to play a crucial role in PVR. Both PDGF and PDGFR-**α** are gaining more attention as novel therapeutic targets.

### 3.2. The Role of PDGF and PDGFR in the Pathogenesis of PVR

In recent decades, vitreous samples from patients undergoing vitrectomy for PVR were found to have elevated concentrations of FGF and PDGF when compared to patients with RD uncomplicated by PVR [69]. PDGF is an abundant regulator of cell growth and division. It plays a central role in blood vessel formation (angiogenesis) [70] and is produced by a plethora of cells, including SM cells, activated macrophages, endothelial cells, and RPE. PDGF is also synthesized, stored, and released by platelets upon activation.

PDGF exists as a dimeric glycoprotein composed of 2 A (-AA) or 2 B (-BB) chains or a combination of the two (-AB). PDGF acts as a chemoattractant and mediator of cellular contraction in RPE cells [71, 72]; it is a potent mitogen for cells of mesenchymal origin, such as smooth muscle and glial cells.

The PDGF signaling network consists of 4 ligands (PDGF-A, PDGF-B, PDGF-C, and PDGF-D) and 2 receptors (PDGFR-**α** and PDGFR-**β**). PDGFRs are classified as tyrosine kinase receptors and are encoded by 2 genes that can homodimerize or heterodimerize to form PDGFR-*αα*, PDGF-**ββ**, and PDGFR-**αβ**. PDGF is mitogenic during early development; during later maturation stages, it has been implicated in cellular differentiation, tissue remodeling, and morphogenesis. PDGF has been shown to direct the proliferation, migration, division, differentiation, and function of a variety of specialized mesenchymal and migratory cell types, especially fibroblasts, during development as well as adulthood [72]. In essence, PDGF allows a cell to skip the G1 (growth) phase in order to divide. Lei et al. found that the presence of PDGF, mainly PDGF-C, in the vitreous cavity was tightly associated with PVR, present in 8/9 PVR patients versus 1/16 patients with other types of retinal disease [73].

The analysis of epiretinal membranes from eyes with PVR showed RPE and Müller cell overexpression of PDGF and PDGFR-*α* [45, 53, 74]. PDGF, with PDGF-C as the predominant isoform, is highly expressed in the vitreous of humans and animals with PVR [75]. PDGF-C is secreted as a latent protein that requires proteolytic processing for activation. Plasmin has been identified as the major PDGF-C processing protease in the vitreous of PVR animals and patients undergoing retinal vitrectomy. The blockade of plasmin prevents the generation of active PDGF-C [55]. 

PDGF-C, together with its receptor PDGFR-*α*, is currently considered as the main contributor to PVR pathology in ocular trauma. PDGFR-*α* has been shown to be more readily activated than PDGFR-**β** and more likely to contribute to PVR [74]. Increased expression of PDGFR-*α* in the retina is associated with the formation of epiretinal membranes and the proliferation of RPE cells and Müller cells [45, 76, 77]. Furthermore, the expression of functional PDGFRs in either RPE or fibroblasts is an essential step for experimental PVR [53, 75, 78]. However, in animal models, cells with no PDGFR-*α* carried a low risk of developing PVR and were able to revert to PVR reexpression upon reestablishment of the wild-type PDGFR genotype. Similarly, blocking PDGFR reduced the potential for PVR development [78]. Nonetheless, recent investigations have shown that blocking PDGF was not sufficient to block PDGFR-*α* activity [79].

Various PDGF isoforms are abundant in the vitreous of patients and experimental animals with PVR but make only a minor contribution to activating PDGFR-*α* and driving experimental PVR. Experimental PVR was found to be dependent on PDGFR-*α* activation, rather than the concentration of PDGF. PEGFR-*α* is also activated by EGF, FGF, insulin, and HGF [75, 78, 79]. Probably indirect activation of PDGFR-*α* by non-PDGF agents is the most important way to activate PVR also by other growth factors.

Vascular endothelial growth factor A (VEGF-A), which mediates neovascularization, competitively blocks PDGF-dependent binding and PDGFR-*α* activation [80]. However, a recent study showed that intravitreal agents that neutralize VEGF-A also inhibit non-PDGF-mediated activation, which protects against PVR [81]. PDGFR-*α* is a tyrosine kinase receptor that requires high levels of intracellular reactive oxygen species. Activation by non-PDGF agents increases intracellular levels of reactive oxygen species (ROS), which in turn activate Src kinase and PDGFR, promoting PVR [81].

Clinical researchers are currently evaluating drugs that target PDGFR-*α* or signaling events required for indirectly activating PDGFR-*α* rather than directly activating PDGF. Antioxidant-directed approaches such as those using N-acetylcysteine or tyrosine kinase inhibitors such as AG1295 or SU9518 could protect against PVR in humans [81–84]. 

### 3.3. Other Growth Factors and Cytokines

TGF-*β* is another growth factor implicated in PVR progression. TGF-*β*_2_ is the most predominant isoform in the posterior segment [85] and is secreted as a latent inactive peptide into the vitreous by epithelial cells of the ciliary body and the lens epithelium. TGF-*β*_2_ is also produced by RPE and Müller cells, fibroblasts, platelets, and macrophages [58]. Similar to PDGF, TGF-*β*_2_ is 3 times more abundant in eyes affected by PVR versus normal eyes [86, 87].

TGF-*β*_2_ is a potent chemoattractant secreted by RPE cells that plays a key role in transforming RPE cells into mesenchymal fibroblastic cells and in inducing type I collagen and extracellular matrix synthesis in RPE cells [88, 89]. Like PDGF, TGF-*β*_2_ was found to increase RPE-mediated retinal contraction. Antibodies against TGF-*β*_2_ and IL-10, an antagonist of TGF-*β*, inhibit the contractility of RPE cells on epiretinal membranes [90]. In vivo experiments have shown that decorin, a naturally occurring TGF-*β* inhibitor, and fasudil, a potent inhibitor of a key downstream mediator of TGF-*β* called Rho-kinase, may reduce fibrosis and RD development [91–94].

Another factor that has been implicated in inflammation and is considered to promote PVR is TNF-*α*, a monocyte-derived cytotoxin. The presence of active TNF-*α* increases serum concentrations of the soluble form of its receptor (sTNF-RI and sTNF-RII), which can be used as a marker of active inflammation [95]. Genetic analysis has identified a single nucleotide polymorphism of the TNF locus that predisposes the eye to PVR [96].

HGF stimulates RPE cell migration and is present at high levels in retinal membranes. It is secreted by macrophages and acts as a multifunctional cytokine on cells of epithelial origin. HGF is also a potent chemoattractant for cultured human RPE cells. Its ability to stimulate cell motility, mitogenesis, and matrix invasion makes it a central player in tissue regeneration and in RPE-related diseases such as PVR [52, 97].

Mounting evidence suggests that chemokines play a role in the inflammatory pathways involved in PVR. Those named most commonly are IL-1*β*, IL-6, IFN-*γ*, and monocyte chemoattractant protein- (MCP-) 1. IL-6 is secreted by T cells and macrophages to stimulate the immune response after trauma, especially burns or other tissue damage leading to inflammation. IL-6 stimulates the proliferation of glial cells and fibroblasts and promotes the synthesis of collagen during wound healing [98]. IL-6 levels are significantly higher in the vitreous and subretinal fluid (SRF) in PVR, particularly posttraumatic PVR [52, 98]. In a recent study, IL-6 levels in the vitreous were found to be predictive for the development of PVR [87].

MMPs are proteolytic enzymes involved in MEC homeostasis; their expression is largely modulated by IL-6. IL6, MMP, and TIMP1 are expressed at high levels in grade B PVR, which involves intense MEC remodeling, [99].

Another cytokine involved in PVR is IFN-*γ*, a dimerized soluble cytokine that is the only member of the type II class of interferons. IFN-*γ* has a variable capacity to stimulate the immune response; this cytokine appears to activate macrophages during the development of PVR. IFN-*γ* levels are 6 times higher in eyes with PVR as compared with control eyes [100].

As mentioned above, the molecular events leading to epiretinal membrane formation in PVR are similar to those occurring in normal wound healing and scar formation [101]. Mononuclear phagocytes play a central role. MCP-1 is implicated in recruiting and directing leukocyte movement [102]. Abu El-Asrar et al. found that MCP-1 is present in the vast majority (76%) of eyes affected by PVR [103].

### 3.4. Emerging Therapeutic Opportunities

Few published studies have investigated the prevention of posttraumatic PVR; surgical management remains the primary mode of therapy. However, it is possible to extend the findings about emerging therapies for the prophylaxis of PVR, the prevention of posttraumatic PVR, on the basis of the molecular mechanisms described above. The most important therapeutic targets in efforts to control the immune response after trauma are Müller and EPR cell proliferation and epiretinal membrane formation.

A recent research on a feline model of RD reported that hyperoxic conditions reduced glutamate cycling dysregulation as well as Müller cell proliferation and transformation [3]. Similar experiments were then conducted in the ground squirrel retina, which is cone-dominated, in contrast to the rod-dominated feline retina [35]. The squirrel study showed a similarly protective effect of oxygen supplementation on photoreceptor degeneration. Providing supplemental oxygen after a diagnosis of RD may help to improve VA recovery after surgery and may reduce the incidence and severity of glial-based complications, such as PVR. Clinical trials with corticosteroids and antiproliferative agents have demonstrated clear success in preventing PVR.

The compounds tested for their ability to prevent PVR include antineoplastic agents, antiproliferative agents, anti-inflammatory agents, antioxidant agents, and anti-growth-factor agents. Current pharmacologic intervention to prevent PVR is principally focused on the use of antiproliferative and anti-inflammatory agents [104]. A number of antiproliferative drugs such as colchicine, daunomycin, alkylphosphocholines, and 5-FU have been tested due to their ability to inhibit the proliferation of human retinal glial cells in vitro. These antiproliferative compounds inhibit non-neural retinal cells, including Müller cells, which can form subretinal membranes that block photoreceptor outer segment regeneration after successful reattachment surgery [105]. One of the most promising antiproliferative candidates is 5-FU; it has been tested in combination with heparin in recent clinical trials. 5-FU acts on DNA synthesis by inhibiting thymidine formation, which inhibits cell proliferation, particularly in fibroblasts. This appears to improve the prognosis for long-term retinal reattachment following the development of PVR in animal models [106, 107]. 

Because 5-FU and low molecular weight heparin (LMWH) are involved in two different aspects of PVR pathogenesis, the two compounds are used together to exert a synergistic effect. Heparin is a naturally occurring complex polysaccharide that is able to bind fibronectin and a range of growth factors involved in the pathogenesis of PVR, such as FGF and PDGF [108]. 

One randomized clinical trial included 174 high-risk patients undergoing primary vitrectomy for RRD who were randomized to receive either 200 *μ*g/mL 5-FU and 5 IU/mL LMWH or placebo. The results showed a significant reduction in the incidence of postoperative PVR and reoperation rates in the patients who received 5-FU and LMWH therapy [109]. Wickham et al. performed a prospective randomized clinical trial that included 641 patients who presented with primary retinal detachment. Patients were treated by either vitrectomy and adjuvant therapy of 5 IU/mL of LMWH and 200 mg/mL of 5-FU or vitrectomy and placebo [110]. These results showed that the use of 5-FU and LMWH did not improve anatomic or visual success rates after 6 months. This discrepancy may stem from the inclusion criteria used for each study: the first study included high-risk patients, the latter included patients with primary RD. Although the efficacy of LMWH with 5-FU infusion during vitrectomy in preventing PVR remains controversial, this combined therapy may be used in the future to treat high-risk patients [111].

Another drug that has been used to inhibit the uncontrolled mitogenic activity of cells at the vitreoretinal interface is daunomycin; it is an anthracycline antibiotic, a topoisomerase inhibitor of DNA and RNA synthesis that arrests cell proliferation and cell migration. This antiproliferative compound inhibits fibroblast and RPE cell proliferation in vitro [112]. Since 1984, daunorubicin has been used for the prophylaxis of idiopathic and traumatic PVR [113]. In a multicenter, prospective, randomized and controlled study that used daunomycin to treat PVR, use of this compound during the vitrectomy increased the rate of reattachment. The evidence for any impact on anatomical success rate and/or visual outcomes was inconclusive [114]. 

In the early nineties, Campochiaro et al. were the first to put in evidence the ability of retinoic acids (RA) in inhibiting RPE cell growth in vitro [115]; subsequently also retrospective and prospective in vivo studies have been conducted [116].

Encouraging results from the use of retinoic acid were published by Chang et al. from a prospective controlled interventional case series of 35 patients affected by retinal detachment complicated with PVR who were randomized to receive either 10 mg oral RA twice daily for 8 weeks postoperatively or placebo. At a one-year postoperative follow-up, the treated group had significantly lower rates of macular pucker formation with higher rates of retinal reattachment [117].

Efforts to inhibit growth factor activity have focused on the tyrosine kinase receptor. Umazume et al. found that dasatinib prevents RPE sheet growth, cell migration, cell proliferation, the epithelial-mesenchymal transition (EMT), and extracellular matrix contraction in a concentration-dependent manner and prevents tractional retinal detachment (TRD) without any detectable toxicity [118]. 

PDGFR-*α* can be activated by PDGF, VEGF, and various other growth factors [78, 119]. VEGF binding to the receptor prevented PVR development in an animal model. 

The apparent mechanism of action of ranibizumab involves the depression of PDGFs, which, at the concentrations present in PVR vitreous, inhibits non-PDGF-mediated activation of PDGF receptor alpha. The inhibition of the receptor by the way of non-PDGF results in a protection for the development of PVR in rabbit models. These preclinical findings suggest that the approaches to neutralize VEGF-A seem to be prophylactic for PVR, but more investigations are needed [81].

Because PVR is thought to be caused by the inflammatory healing process, intravitreal corticosteroids may be of use for treatment. These compounds exert their therapeutic action by limiting BRB breakdown, reducing neutrophil transmigration, inhibiting fibroblast proliferation, suppressing macrophage recruitment, limiting leucocyte migration, decreasing cytokine production, and reducing the formation of granulation tissue [120].

Corticosteroids inhibit the proliferation of fibroblasts, RPE cells, and RPE-transformed myofibroblasts that are responsible for the contractile properties of PVR membranes [121, 122]. Steroids also seem to interfere with the recruitment of macrophages to the site of a lesion and may block the action of monocyte/migration inhibitory factors (MIFs) [123]. 

These drugs are applied topically as eye drops, locally by subconjunctival, peribulbar, or retrobulbar injection, and systemically via oral, intravenous, and intramuscular routes. Numerous experimental studies conducted on animal models have demonstrated the benefits of the intravitreal administration of triamcinolone [124]. Despite this success in animal models, the same positive results have not been achieved in human studies.

Encouraging results regarding the use of triamcinolone acetonide emerged from a study conducted by Jonas et al. The authors demonstrated that the intravitreal injection of crystalline cortisone reduces postoperative intraocular inflammation. However, the mean follow-up period adopted in this study was less than 2 months, which reduces the validity of the results [125]. Despite the potential benefits, the intravitreal injection of triamcinolone acetonide is associated with side effects, including glaucoma and cataract, so recent research in this area has focused on the use of dexamethasone. A recent study conducted by Bali et al. showed that the subconjunctival injection of dexamethasone prior to surgery decreased the extent of postoperative BRB breakdown as measured by laser flare photometry 1 week postoperatively [122]. 

In this regard, we take the opportunity to report an important recent study in which Hoerster et al. evaluated the anterior chamber aqueous flare with laser flare photometry and found that it is a strong preoperative predictor for PVR in eyes with RD [126].

The disadvantage of using dexamethasone is the compound's short half-life, which has led to the development of long-acting intravitreal dexamethasone implants [127].

The antioxidant compounds represent one last class of drugs under investigation. As demonstrated by Lei and Kazlauskas, the indirect activation of PDGFR triggers signaling events leading to PVR [83]. Non-PDGF growth factors can increase intracellular concentrations of reactive oxygen species (ROS), leading to PDGFR activation. Lei et al. tested whether an antioxidant such as N-acetylcysteine (NAC) was able to prevent the accumulation of ROS and thereby block PDGFR activation. A 10 mmol/L-dose of NAC suppressed PDGFR-*α* activation and protected against RD in a rabbit model. Although NAC did not prevent the formation of an epiretinal membrane, the compound did limit the extent of vitreous-driven contraction [128]. Antioxidants may prevent detachments after retinal surgery and should be considered for use in combination with other therapeutic approaches.

## 4. Conclusions

Although the exact impetus for proliferation remains unknown, there is compelling evidence that posttraumatic PVR is similar to wound healing in terms of the inflammation, proliferation, and remodeling involved. The greatest challenge is to identify a pharmacological approach and adjuvant surgery that could be truly prophylactic for the development of PVR. 

Various pharmacological agents have demonstrated potential in reducing postoperative PVR risks, including intravitreal LMWH, 5-FU, daunomycin, and anti-VEGF drugs. Clinical reports have suggested that either systemic or intravitreal corticosteroids may be useful in attenuating PVR gravity by limiting BRB breakdown. However, many clinical trials have shown inconclusive results; none of these agents has been shown to be decisive in preventing PVR after surgery.

Our knowledge about the pathogenesis of PVR has improved over recent decades. The introduction of immune cells into the vitreous cavity, as is the case in penetrating ocular trauma, triggers the production of growth factors and cytokines that come in contact with intraretinal Müller and RPE cells. It is widely accepted that growth factors and cytokines, including PDGFs, HGF, TNF*α*, and bFGF, drive the cellular responses intrinsic to PVR. These cytokines and growth factors promote an environment of cell transdifferentiation, migration, and proliferation that allows for expansion of the extracellular matrix. As this scaffold forms, it may physically attach to the retina. Subsequent contraction causes wrinkling, shortening, and tearing of the retinal tissue, otherwise known as PVR. 

The process involves a host of cytokines and growth factors. To our knowledge, none seem to be indispensable for disease onset or progression. However, these pathways appear to converge at the steps necessary for the expression and activation of PDGFR-*α*, which seem to be crucial in the development of PVR. 

In addition to the PDGFs, all of the other growth factors mentioned above stimulate the expression and activation of PDGFR-*α* on the surface of RPE cells, Müller cells, glial cells, and fibroblasts. The activity of this receptor promotes transdifferentiation, migration, proliferation, survival, the formation of extracellular matrix, membrane formation, and contraction. A combination therapy that could block all of these agents would be an ideal addition to the arsenal currently used to prevent PVR. 

When used in combination with other tyrosine kinase inhibitors, the antioxidant NAC prevents tractional RD in animal models by blocking non-PDGF growth factor-mediated PDGFR-*α* activation. A recent study showed that a cocktail of neutralizing reagents targeted to multiple growth factors and cytokines was able to reduce PVR development. Antibodies against PDGF, EGF, FGF-2, IFN-*γ*, IL-8, TGF-*α*, VEGF, TGF-*β*, HGF, and IGF-1 to IGF-12 were effective in preventing RD in a rabbit model [129].

In the future, novel therapeutic agents could enhance functional recovery after RD by limiting cellular proliferation. A combined therapy involving oxygen supplementation, a cocktail of neutralizing reagents, and tyrosine kinase inhibitors would target intracellular and extracellular activation of PDFGR-*α*, thereby protecting against PVR. Current investigations into the pathobiological and pathophysiological mechanisms involved are increasing the possibilities for management. It is hoped that our treatment strategies will evolve and become even more effective in achieving complete vision recovery. 
